# Sleep deprivation and recovery sleep affect healthy male resident’s pain sensitivity and oxidative stress markers: The medial prefrontal cortex may play a role in sleep deprivation model

**DOI:** 10.3389/fnmol.2022.937468

**Published:** 2022-08-18

**Authors:** Shuhan Chen, Yanle Xie, Yize Li, Xiaochong Fan, Fei Xing, Yuanyuan Mao, Na Xing, Jingping Wang, Jianjun Yang, Zhongyu Wang, Jingjing Yuan

**Affiliations:** ^1^Department of Anesthesiology, Pain and Perioperative Medicine, The First Affiliated Hospital of Zhengzhou University, Zhengzhou, China; ^2^Henan Province International Joint Laboratory of Pain, Cognition and Emotion, Zhengzhou, China; ^3^Department of Anesthesiology, Tianjin Research Institute of Anesthesiology, Tianjin Medical University General Hospital, Tianjin, China; ^4^Massachusetts General Hospital Department of Anesthesia, Critical Care and Pain Medicine, Harvard Medical School, Boston, MA, United States

**Keywords:** sleep deprivation, pain, oxidative stress, recovery sleep, night shift

## Abstract

Sleep is essential for the body’s repair and recovery, including supplementation with antioxidants to maintain the balance of the body’s redox state. Changes in sleep patterns have been reported to alter this repair function, leading to changes in disease susceptibility or behavior. Here, we recruited healthy male physicians and measured the extent of the effect of overnight sleep deprivation (SD) and recovery sleep (RS) on nociceptive thresholds and systemic (plasma-derived) redox metabolism, namely, the major antioxidants glutathione (GSH), catalase (CAT), malondialdehyde (MDA), and superoxide dismutase (SOD). Twenty subjects underwent morning measurements before and after overnight total SD and RS. We found that one night of SD can lead to increased nociceptive hypersensitivity and the pain scores of the Numerical Rating Scale (NRS) and that one night of RS can reverse this change. Pre- and post-SD biochemical assays showed an increase in MDA levels and CAT activity and a decrease in GSH levels and SOD activity after overnight SD. Biochemical assays before and after RS showed a partial recovery of MDA levels and a basic recovery of CAT activity to baseline levels. An animal study showed that SD can cause a significant decrease in the paw withdrawal threshold and paw withdrawal latency in rats, and after 4 days of unrestricted sleep, pain thresholds can be restored to normal. We performed proteomics in the rat medial prefrontal cortex (mPFC) and showed that 37 proteins were significantly altered after 6 days of SD. Current findings showed that SD causes nociceptive hyperalgesia and oxidative stress, and RS can restore pain thresholds and repair oxidative stress damage in the body. However, one night of RS is not enough for repairing oxidative stress damage in the human body.

## Introduction

Chronic pain is a major health problem worldwide (Cohen et al., 2021). sleep might be relevant for predicting both the onset and the resolution of pain (Aili et al., 2015). The deprivation or the disturbance of sleep enhances pain sensitivity (Lautenbacher et al., 2006). Previous studies suggest that sleep deprivation (SD), or sleep loss, results in augmented pain response in healthy subjects (Roehrs et al., 2006; Azevedo et al., 2011). Experimental studies highlight the profound impact of sleep disruptions on pain, suggesting that SD leads to hyperalgesic pain changes, while chronic pains are often accompanied by sleep disturbances (McBeth et al., 2015; Sivertsen et al., 2015). In chronic pain, the activity of the medial prefrontal cortex (mPFC), a brain region critical for executive function and working memory, is severely impaired. The major extracortical sources of excitatory input to the mPFC come from the thalamus, the hippocampus, and the amygdala, which enables the mPFC to integrate multiple streams of information required for the cognitive control of pain, namely, sensory information, context, and emotional salience (Jefferson et al., 2021). One study found that one night of total SD impaired descending pain pathways and sensitized peripheral pathways to cold and pressure pain (Staffe et al., 2019). Chronic SD may alter the habituation and sensitization of pain, thereby increasing susceptibility to pain (Simpson et al., 2018). At the same time, some studies showed that undisturbed sleep and recovery sleep (RS) may in turn help the pain system return to normal overnight. The question of whether RS helps to reset occurring pain changes has gained special interest. Roehrs et al. (2012) have demonstrated that, in healthy sleep-deprived subjects, improved sleep quality/quantity leads to decreased pain sensitivity thereafter.

Night shift work has a critical effect on sleep, and it is inevitable for anesthesiologists to work on the night shift. Many anesthesiologists even work through the night when they are on the night shift. Extended shift durations and interrupted sleep are integral parts of an anesthesiologist’s professional life (Tucker and Byrne, 2014). Despite recommendations in the United States and in Europe to limit work hours, the amount worked can still not only lead to acute and chronic SD but may also present a potential hazard for doctors’ health. Anesthesiologists experience disruptions in their circadian rhythms as a result of working through the night. Sleep disruption or SD is thought to increase oxidative stress, defined as an imbalance in the normal balance between reactive oxygen species formation and antioxidant defense mechanisms. Anesthesiologists switching between night shifts and day shifts, or taking shifts off, are at risk of circadian rhythm disruptions. Circadian rhythms provide the regulation of protein expression in response to oxidative stress (Wilking et al., 2013). Chronic oxidative stress caused by sleep disruption can lead to chronic systemic low-grade inflammation (Besedovsky et al., 2019) and long-term disruption of circadian rhythms (Morris et al., 2018).

Many studies have shown a close link between the mPFC and sleep. Up to 12 circadian core clock genes are altered by SD in mice mPFC (Guo et al., 2019). Acute paradoxical deprivation leads to microglia activation and inflammatory responses in the prefrontal cortex of mice (Liu et al., 2022), which can also cause oxidative stress and lead to a decrease in glutathione (GSH) levels (Kanazawa et al., 2016). SD selectively enhances the expression and activity of prefrontal cortical 5-α reductase in rats, thereby affecting their mental status (Frau et al., 2017). Also, several pieces of clinical evidence showed that the morphology of the mPFC is altered in humans after SD (Mullin et al., 2013; Elvsåshagen et al., 2017; Feng et al., 2018).

Given the above evidence, we believe that oxidative stress and pain threshold sensitivity may be increased after one night of SD, which may be ameliorated by one night of RS. Therefore, the purpose of this study was to evaluate (1) the effect of SD and RS on the pain sensitivity of participants and animals, (2) the extent of overnight total SD and one night of RS on systemic redox of participants, and (3) changes and effects of mPFC in rats during SD.

## Materials and methods

### Participants

The study was approved by the ethics committee of the First Affiliated Hospital of Zhengzhou University (2021-KY-1149-004). The trial was registered at the Chinese Clinical Trial Registry (ChiCTR2200058546). Anesthesia residents of the First Affiliated Hospital of Zhengzhou University, years 18 to 44, were invited to participate in the study. The recruited subjects were all anesthesiologists working in the First Affiliated Hospital of Zhengzhou University. We recruited 24 male volunteers within the hospital department, four of whom were excluded due to ineligibility, so, a total of 20 participants were selected for the study. The mean age of male participants was 26 ± 2 years (range 18∼44 years). Participants were screened to ensure good health, including the documentation of habitual nightly sleep duration between 7 and 9 h; the absence of sleep disorders based on questionnaires; the absence of any psychiatric disorders or chronic health disorders (e.g., hypertension, chronic pain disorders); the absence of any acute health problems (e.g., broken arm, cold, or flu); the absence of regular medication use; the absence of dietary supplements use, namely, vitamins and cod liver oil; and body mass index (BMI) <27.5 kg/m^2^. Baseline demographic measures were collected. Participants were not allowed to consume alcohol, chocolate, or caffeine for 72 h before and during the study. Strenuous exercise and napping were prohibited.

In the pilot trial, subjects’ pressure pain thresholds (PPTs) were measured at baseline, after SD, and after RS. The PPTs at the three timespoints were 265, 160, and 250 mg, respectively, with a standard deviation of 80. While setting α to 0.05 (bilateral), β to 0.10, and the number of groups k to 3, the minimum sample size of 13 cases was calculated. Twenty-four patients were recruited to ensure complete data collection, considering the trial’s dropout rate, exclusion rate, and compliance.

#### Procedure

**FIGURE 1:**
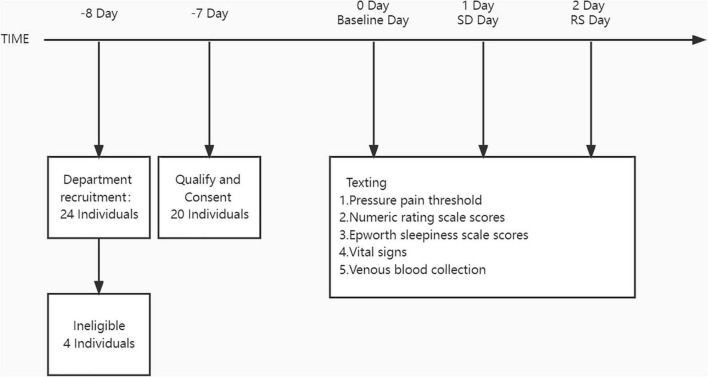
Study protocols.

The study was conducted in the First Affiliated Hospital of Zhengzhou University. Figure 1 depicts the participants’ schedules and study protocols. Participants were not divided into groups and were self-controlled. Intensive 24-h recordings for 3 days were obtained (baseline night, SD night, and RS night). These intensive recording periods included major signs check, blood sampling, Epworth sleepiness scale (ESS), and pain evaluation.

During SD, the participants were permitted to engage in non-stressful activities (e. g., talking, board games, etc.) or were only allowed to drink water and were forbidden to take food or drink or do strenuous exercise. During the SD evening, the participants were not allowed to sleep after 8 p.m. overnight. Participants were continuously monitored by researchers and smart watches throughout the night (Huawei Watch Fit 6, FRA-B19, Huawei Technologies Co., Ltd.).

#### Blood sample collection

To measure the levels of malondialdehyde (MDA), catalase (CAT), GSH, and superoxide dismutase (SOD), 3 mL of blood was taken through venipuncture and collected into ethylenediaminetetraacetic acid (EDTA) coated tubes. The tubes were immediately centrifuged for 10 min at 1,500 × *g* at 4°C after collection. The plasma was then apportioned into 0.5 mL aliquots and stored at −20°C until analyses were conducted.

#### Pressure pain thresholds of the volunteers

Pressure pain thresholds of participants were evaluated by using von Frey filaments. A von Frey hair weighing 60 to 300 g was used. The filaments were presented in the increasing order of strength, perpendicular to the surface of the back of the subject’s hand, with enough force to cause it to bend slightly. Until the subjects reported a pain response and stopped, the weight of the filaments was then recorded as the subject’s pain threshold. Pain sensitivity were assessed on the Numerical Rating Scale (NRS) every morning after each night. Taking circadian rhythm into account, these recordings were assessed by 20 anesthesiologists between 7:30 and 8:00 a.m. for 3 days. The research environment was tightly controlled. The investigators conducting the tests were blinded to the group.

### Animals

This study was approved by the Ethics Committee of Zhengzhou University and was performed in accordance with the Guide for the Care and Use of Laboratory Animals from the National Institutes of Health, United States. Adult male Sprague-Dawley rats [total *n* = 26; SPF (Beijing) biotechnology co., Ltd.], weighing 220∼250 g on arrival, were initially housed in pairs in an animal colony room under a 12-h light/dark cycle (lights on at 7:00 a.m.) at 23 ± 1°C, with food and water available *ad libitum*. All rats were allowed 2 weeks to acclimate to the surroundings before the beginning of the experiments.

#### Animal treatment

Rats were divided into the control group (sham, *n* = 8) and the SD group (SDR, *n* = 8). Rats were subjected to 6 h of SD from 9 a.m. to 3 p.m. per day for 6 days. An SD rat model was established using a modified multiplatform water environment method (Gao et al., 2019) and was provided food and drinking water. Control rats were allowed to sleep in cages without restriction. After 6 days of SD, the rats in both groups slept unrestrictedly, which was called RS.

#### Paw withdrawal threshold in rats

Mechanical allodynia was evaluated by determining PWT by using the up and down method with von Frey filaments. The rats were placed on a barbed wire platform and allowed to acclimate for 30 min before the test. A von Frey hair weighing 2.0 to 26.0 g was used. Then, the filaments were presented, in ascending order of strength, perpendicular to the plantar surface with sufficient force to cause slight bending against the paw. Paw withdrawal, flinching, or paw licking was considered a positive response. After a response, the filament of the next lower force was applied. In the absence of a response, the filament of the next greater force was applied. The tactile stimulus producing a 50% likelihood of withdrawal was determined using the “up-down” method. The investigators performing the behavioral tests were blinded to the group.

#### Paw withdrawal latency in rats

The Model 336 Analgesia Meter (IITC Inc. Life Science Instruments, Woodland Hills, CA, United States) was used to measure PWL to noxious heat. The rats were placed on an elevated glass plate and allowed to acclimatize for 30 min before the test. A radiant thermal stimulator was focused on the plantar surface of the hind paw through the glass plate. The nociceptive endpoint of the radiant heat test was characteristic movements like lifting or licking the hind paw, and the time to the endpoint was considered PWL. To avoid tissue damage, 20 s was used as a cutoff time. Each animal had five trials with a 10-min interval per side. The investigators conducting the behavioral tests were blinded to the group.

### Biochemical analysis of oxidative stress

As described in the study protocol, plasma samples were collected upon completing all tests. The following parameters were measured: (1) MDA concentration, (2) GSH concentration, (3) SOD activity, and (4) CAT activity.

The MDA concentration in the plasma was determined using a commercially available kit (Nanjing Jiancheng Bioengineering Institute, Nanjing, China) based on thiobarbituric acid (TBA) reactivity. The test principle is that MDA in the degradation product of lipid peroxide can be combined with thiobarbituric acid to form a red product with a maximum absorption peak at 532 nm.

The GSH concentration in the plasma was determined by the microplate method using a commercially available kit (Nanjing Jiancheng Bioengineering Institute, Nanjing, China). Reduced GSH can react with 5,5-Dithio-bis-(2-nitrobenzoic acid) (DTNB) to produce a yellow compound, and the content of reduced GSH can be determined by colorimetric quantification at 405 nm.

The SOD activity in the plasma was determined using a commercial kit (Nanjing Jiancheng Bioengineering Institute, Nanjing, China) based on the hydroxylamine method. When SOD is contained in the tested sample, it has a specific inhibitory effect on superoxide anion free radical, reducing the formation of nitrite, and the absorbance value of the determination tube is lower than that of the care. SOD activity in the tested sample can be calculated by formula calculation.

The CAT activity in plasma was determined by an ammonium molybdate assay using a commercial kit (Nanjing Jiancheng Bioengineering Institute, Nanjing, China). The decomposition of H_2_O_2_ by CAT can be stopped rapidly by adding ammonium molybdate, and the remaining H_2_O_2_ reacts with ammonium molybdate to form a yellowish complex. The activity of CAT can be calculated by measuring its change at 405 nm.

### Proteomic detection and analysis

#### Protein extraction and sample preparation

Rats were anesthetized with chloral hydrate and mPFC was removed on ice. We performed five biological replicates for each group of rats. The removed tissue was rapidly frozen in liquid nitrogen and then stored at −80°C. RIPA lysate was mixed with PMSF (Beijing Solarbio Science & Technology Co., Ltd) to remove the frozen tissue, and 1,000 μL of the lysate was added to the mix and sonicated in an ice bath for 5 min until the tissue fully dissolved. The mixed solution was centrifuged at 14,000 × g for 15 min at 4°C to obtain the sample lysate. Then, the lysate was subjected to bicinchoninic acid (BCA) quantification, acetone precipitation, protein re-solubilization, protein reduction, protein alkylation, proteolysis, sodium deoxycholate (SDC) removal, peptide desalting, and nano ultra-performance liquid chromatography (UPLC) separation to obtain the final sample preparation solution.

#### Label-free quantitative and liquid chromatography-mass spectrometry/mass spectrometry analysis

The sample preparation solution was analyzed by liquid chromatography-mass spectrometry/mass spectrometry (LC-MS/MS). The Q Exactive (Thermo Fisher Scientific, Inc., Shanghai, China) was used to perform LC-MS/MS on the samples to be tested, and each sample was detected for 120 min to obtain the detection data.

#### Database search

For the obtained data, raw files were processed using MaxQuant (2.0.1.0). The protein database was obtained from the UNIPROT database (uniprot-proteome-rat-2021.2).

Protein sequences and their reverse decoy sequences were used for both MaxQuant search libraries. The quantification type was LFQ containing match between run; trypsin was set as a specific endonuclease with a maximum of 2 missed cut sites, oxidation (M) and acetyl (protein N-term) were set as variable modifications, and carbamidomethyl (C) was fixed modification with maximum variable modifications of 5. Peptide and protein level FDR was 0.01. FDR was 0.01 for both peptide and protein levels, and unique peptides without variable modifications were used for quantification. LFQ is used to analyze the mass spectrum data generated during the large-scale identification of proteins by using liquid mass spectrometry technology to compare the signal intensity of corresponding peptides in different samples so as to carry out the relative quantification of proteins corresponding to peptides.

#### Bioinformatics annotation

The normalized quantitative results were then statistically analyzed to obtain the corresponding differentially expressed proteins. Subsequent gene ontology (GO), Kyoto encyclopedia of genes and genomes (KEGG) pathway, protein interaction analysis, and demonstration were performed. Molecular functions, cellular components, and biological processes of GO were analyzed. The KEGG online service tools were used to annotate the protein descriptions. Pathways were classified according to the KEGG website, and further cluster analysis was performed to explore potential connections and differences between specific functions.

### Statistics

For the biochemical indicators and nociceptive test data of the subjects at three-time points, the one-way ANOVA was used for the data that met the normal distribution, and the Kruskal–Wallis test was used to compare the difference of the medians for the data that did not meet the normal distribution. For the pairwise comparisons among three groups, the continuous data satisfied the normal distribution and the homogeneity of variance test used LSD, and the non-parametric test for the pairwise comparisons used the Kruskal–Wallis *H*-test. Behavioral data of rats before and after SD were analyzed by a two-way ANOVA, and Sidak’s multiple comparisons test was used for pairwise comparison of time points. All reported *p*-values are two-tailed with an *a priori* significance level of *P* < 0.05. All statistical analyses of participants were performed using SPSS 24 (SPSS Statistics, IBM, Armonk, NY, United States). Data analysis of rats and graphing software using Prism GraphPad Prism 8 were performed using GraphPad Software Inc.

## Results

**FIGURE 2:**
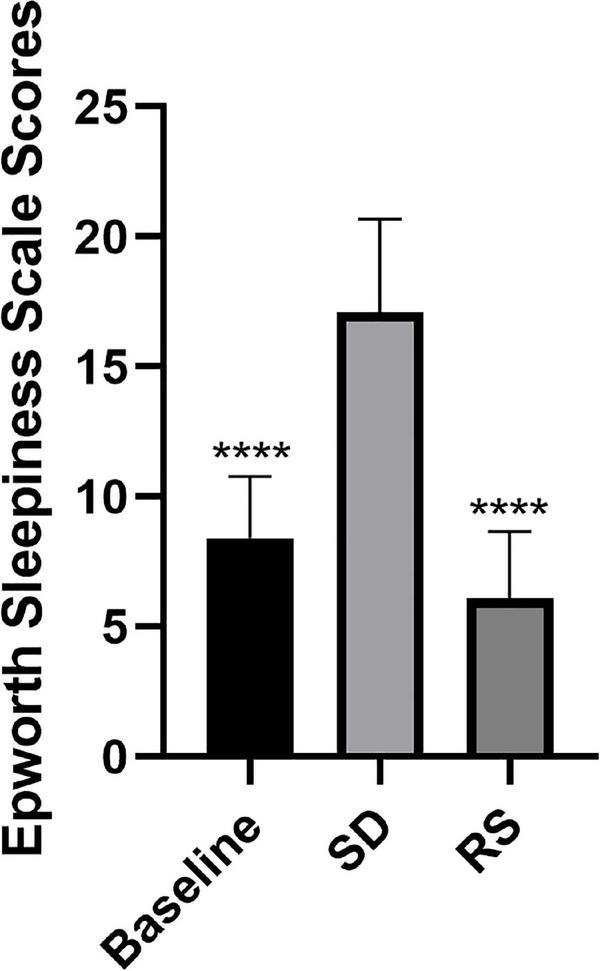
Epworth sleepiness scale scores. Subjects were scored on the Epworth sleepiness scale at three time points. Sleep deprivation significantly increased subjects’ daytime sleepiness compared to baseline, and this was significantly reduced after recovery sleep. ****P < 0.0001 compared to SD.

A total of 20 volunteers, mainly unmarried (*n* = 15 or 75%) men with a BMI of 24.2 kg m^–2^ (standard deviation = 3.8), were recruited for this study. The mean age of the study participants was 26 years (standard deviation = 2) and the mean work experience was 2 (1 to 3) years. Participants’ major signs before and after SD and RS are shown in Table 1. We also recorded ESS scores at these three-time points (Figure 2). One night of SD could significantly increase the degree of drowsiness of participants during the day and significantly decrease the degree of drowsiness after experiencing unrestricted RS.

**TABLE 1:** Participants’ vital signs.

	Baseline (*n* = 20)	SD (*n* = 20)	RS (*n* = 20)	*F*	Overall significance (*P*-value)
Heart rate (times per minute)	82 (76 to 87)	81 (70 to 90)	82 (78 to 87)		0.939
SpO_2_ (%)	100 (99 to 100)	100 (99 to 100)	99 (99 to 100)		0.454
Mean artery Pressure (mmHg)	93.6 ± 7.6	94.5 ± 7.9	93.1 ± 7.7	0.998	0.857

### Sleep deprivation led to nociceptive hypersensitivity

**FIGURE 3:**
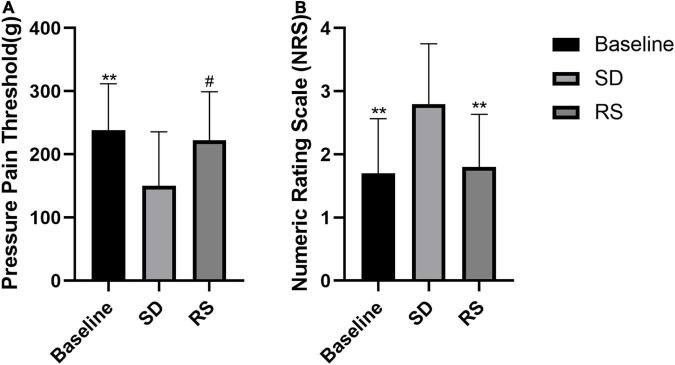
Pressure pain threshold **(A)** and NRS subjective pain scores **(B)**. Subjects’ changes in pressure pain threshold and subjective NRS scores. Sleep deprivation (SD) significantly increased perceived pain compared to baseline, and this nociceptive hyperalgesia was significantly reduced after recovery sleep (RS). ^#^*P* < 0.05 compared to SD. **P < 0.01 compared to SD.

Among participants, we measured nociceptive mechanical pain thresholds and NRS subjective pain scores at baseline, after one night of SD, and after one night of RS (Figure 3). We found that participants’ nociceptive pain thresholds dropped significantly after one night of SD and that one night of RS reversed this hyperalgesia. The NRS scores increased significantly after one night of SD and recovered with one night of RS.

**FIGURE 4:**
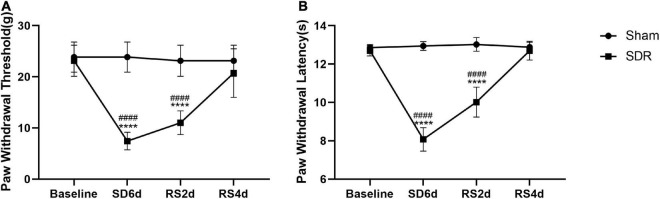
Paw withdrawal threshold **(A)** and paw withdrawal latency **(B)** in rats. Rats 1–8 in the Sham group and 1–8 in the SDR group. The PWT and PWL of rats decreased significantly after 6 days of SD, and the PWL recovered after 2 days of RS compared to 6 days of SD, and both PWT and PWL returned to baseline levels after 4 days of RS. ****P < 0.0001 compared to the sham group. ^####^*P* < 0.0001 compared with baseline and RS4d in the SDR group.

We examined nociceptive thresholds after SD in rats. In the evoked nociceptive tests, compared with the sham group, the SDR group showed significant reductions in both PWT and PWL at 6 days after SD. Our experiments showed a partial recovery of thermal nociceptive sensitization in rats after 2 days of RS and a trend toward the recovery of mechanical nociceptive sensitization without statistical significance. On the fourth day of RS, mechanical nociceptive sensitization and thermal nociceptive sensitization in the SDR group rats returned to baseline (Figure 4).

### Biochemical measures

**FIGURE 5:**
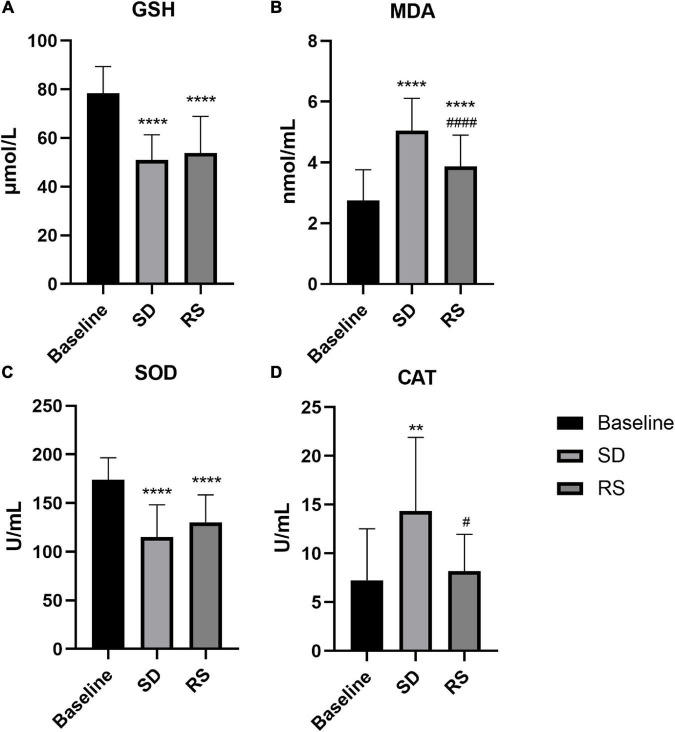
Plasma GSH **(A)**, MDA **(B)**, SOD **(C)**, and CAT **(D)** levels or activity measurements before and after SD and RS. **P < 0.01 compared to Baseline, ****P < 0.0001 compared to Baseline, ^#^*P* < 0.05 compared to SD, ^####^*P* < 0.0001 compared to SD.

Plasma-derived biochemical measures before and after SD are shown in Figure 5. Relative to baseline (mean = 2.8, standard deviation = 1.0 nmol/mL), the MDA levels were significantly increased following one night of SD (mean = 5.0, standard deviation = 1.0 nmol/mL, *P* ≤ 0.0001). The MDA levels partially recovered after RS (mean = 3.9, standard deviation = 1.0 nmol/mL), increasing from baseline (*P* = 0.001), and decreasing from one night of SD (*P* = 0.001). After SD, CAT (12.5 [8.0 to 21.3] U/mL) levels were also significantly increased relative to baseline (5.3 [3.1 to 9.5] U/mL, *P* = 0.001). CAT levels after RS (8.1 [5.5 to 10.9] U/mL) were almost restored to the baseline level (*P* = 0.844) and decreased significantly compared with SD (*P* = 0.038). On the contrary, SD resulted in reduced GSH (mean = 51.0, standard deviation = 10.3 μmol/L) levels that were reduced relative to baseline (mean = 78.4, standard deviation = 11.0 μmol/L, *P* ≤ 0.0001). The level of GSH (mean = 53.9, standard deviation = 15.0 μmol/L) after sleep recovery increased slightly compared with SD, which was not statistically significant (*P* = 0.470), and decreased significantly compared with baseline (*P* ≤ 0.0001). In parallel to this result, SOD levels were also lower after SD (mean = 115.2, standard deviation = 33.1 U/mL) compared with baseline (mean = 174.0, standard deviation = 22.6 U/mL, *P* ≤ 0.0001). There was no significant recovery of the SOD level after RS compared to SD (*P* = 0.095). Compared with baseline, the SOD (mean = 130.4, standard deviation = 28.2 U/mL) levels decreased significantly after RS (*P* < 0.0001).

### Proteomics of medial prefrontal cortex in rats with sleep deprivation

We removed mPFC from the rats after SD for proteomic, and the following results were determined.

#### Medial prefrontal cortex changes in rats treated with sleep deprivation

**FIGURE 6:**
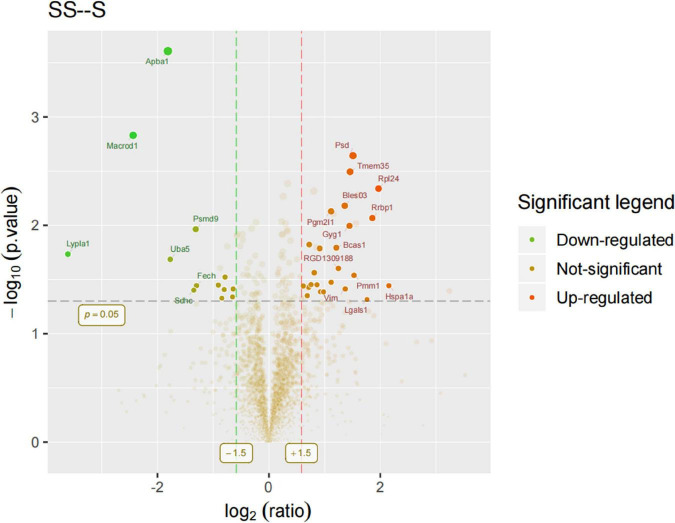
Volcano diagram: Rats 9–14 in the sham group and 9–13 in the SDR group. Differentially expressed proteins were displayed in the volcano diagram. *Y*-axis: -log10 (*p*-value); *X*-axis: log2 (ratio). The dots that lie beyond the two vertical boundaries and above the horizontal boundaries represent proteins that differ significantly. Clear spots in salient regions mean that these proteins do not meet the other conditions.

In order to identify mPFC proteins’ abundances in SD, we searched the raw data in LC-MS/MS databases and got a core set of 260 quantified proteins. Based on the differences in the protein ratios of 260 proteins between the different study groups, we identified 37 potentially differentially expressed proteins associated with SD. Of these 37 proteins, 24 proteins were increased in the SDR group compared to the sham group, while 13 proteins were decreased in the SDR group, and the expression of these proteins differed between experimental groups. Differentially expressed proteins are displayed in the volcano diagram in Figure 6.

#### Functional analysis of the differential proteins associated with sleep deprivation

**FIGURE 7:**
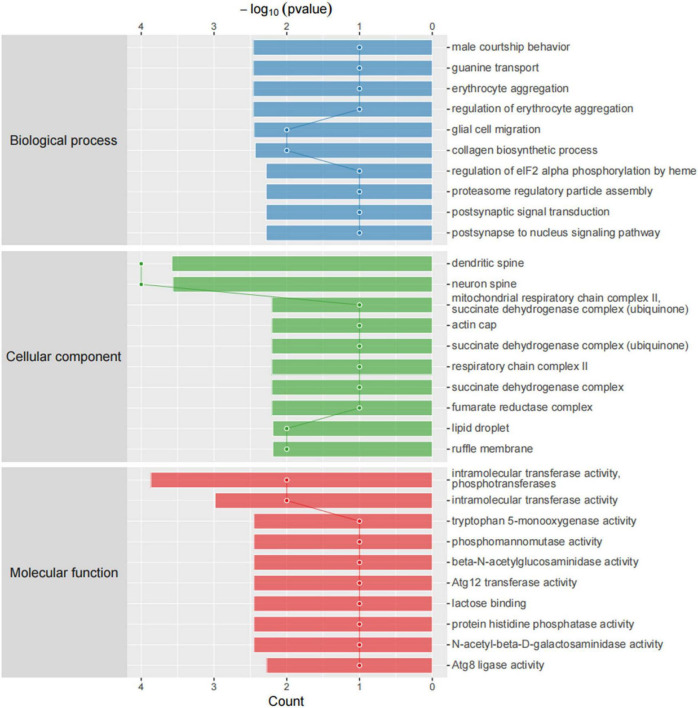
Gene ontology (GO) cellular components. Top axis is -log10 (*p*-values) and bottom axis is gene count. The ontology covers three domains: biological process, cellular component, and molecular function.

**FIGURE 8:**
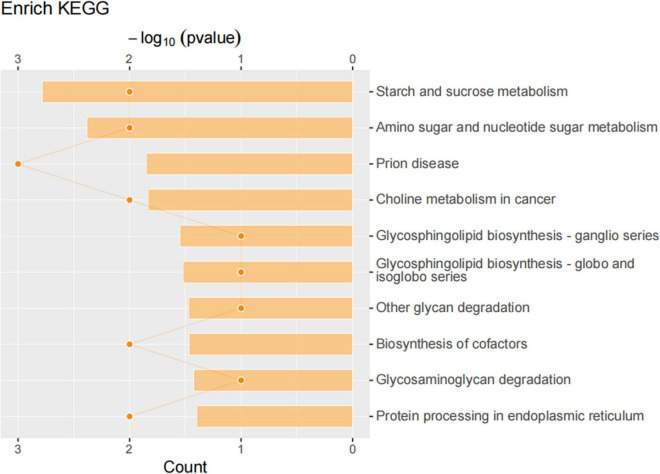
Kyoto Encyclopedia of Genes and Genomes (KEGG) pathway analysis of differentially expressed proteins. KEGG pathway annotation was performed on the selected differentially expressed proteins to analyze and determine the most important metabolic and signal transduction pathways involved in the differentially expressed proteins.

We then compared differentially expressed proteins between the study groups through an enrichment analysis of gene ontology biological processes and identified key biological processes and potential pathways that may distinguish the SDR group from the sham group. The cellular processes represented by the mPFC proteome mainly include guanine transport, glial cell migration, collagen biosynthetic process, erythrocyte aggregation and regulation, post-synaptic signal transduction, and post-synapse to the nucleus signaling pathway. The enrichment analysis of GO cellular components (Figure 7) showed that these proteins were mainly localized in the dendritic spine, the neuron spine, the respiratory chain complex II, and the lipid droplet. These active factors participated in starch and sucrose metabolism, amino sugar and nucleotide sugar metabolism, and the biosynthesis of cofactors, as described in KEGG pathway mapping (Figure 8). GO enrichment analysis showed that tryptophan 5-monooxygenase activity induced changes in molecular function during SD. As a redox substrate, tryptophan 5-monooxygenase is an important enzyme in the synthesis of 5-hydroxytryptamine and is also involved in the synthesis of melatonin. Melatonin is secreted by the brain’s pineal gland, and its secretion has a distinct circadian rhythm regulation and antioxidant effects (Zhang and Zhang, 2014).

#### Network analysis identifies sleep deprivation-related specific protein network in medial prefrontal cortex

**FIGURE 9:**
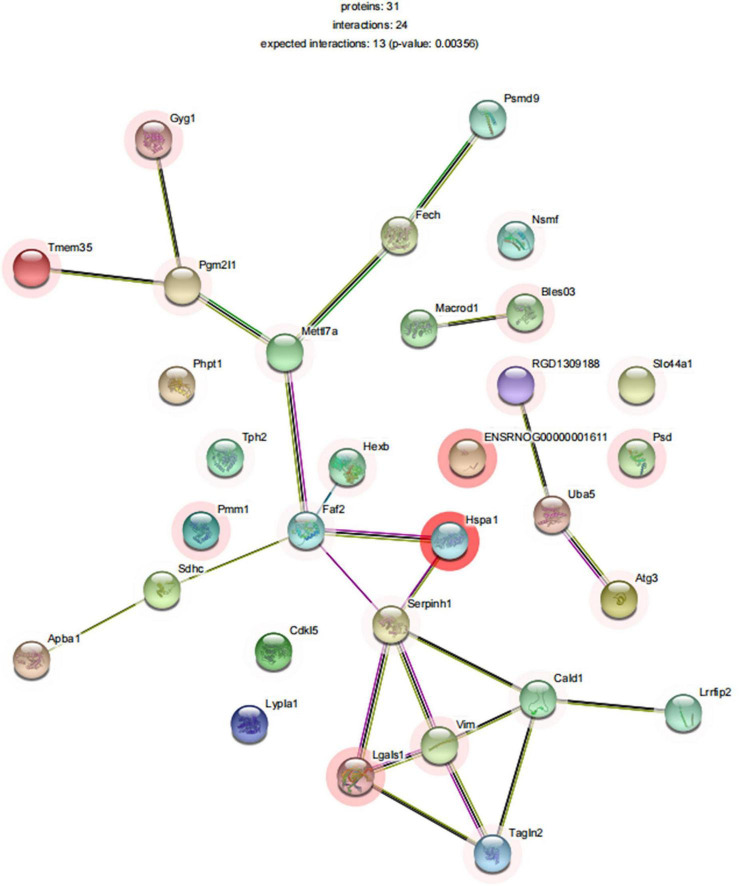
STRINGdb protein–protein network enrichment analysis. The protein–protein interaction network of significant proteins is shown. STRING database integrates various information from curated databases that were experimentally determined; gene neighborhood, gene fusions, and gene co-occurrence; text mining, co-expression, and protein homology.

To explore the relationship between differentially expressed proteins associated with SD, we performed a gene network analysis of differentially expressed proteins in mPFC to identify specific proteins associated with SD, as seen in Figure 9. We analyzed the significantly different proteins expressed in mPFC of rats in the SDR group and the sham group, in an attempt to explore their connection to biological processes or molecular functions. Among them, METTL7A was significantly downregulated in neuropathic pain rats (Gong et al., 2021) and showed a decreasing trend in our SD rats. Bi-allelic PGM2L1 mutations are associated with a neurodevelopmental disorder (Morava et al., 2021). Galectin-1 (ALGAS1) is highly expressed at the sites of infection and inflammation and plays a role in regulating cell differentiation, proliferation, and apoptosis (Méndez-Huergo et al., 2017). Furthermore, pro-inflammatory cytokines in ALGAS1^–/–^ mice were significantly downregulated in the liver tissue (Baek et al., 2021), consistent with our finding that ALGAS1 is significantly upregulated in SD rats. MacroD1 modulates mitochondrial function and DNA damage (Agnew et al., 2018) and can further exacerbate the inflammatory response induced by lipopolysaccharide stimulation (Zang et al., 2018).

## Discussion

Night shifts are very common among clinicians, and anesthesiologists inevitably work night shifts, and they often stay up late on duty in SD. Sleep plays a critical role in regulating cognitive performance and productivity. This is especially true for anesthesiologists and intensivists who often work in shifts (Ariès and Lamblin, 2020). Studies demonstrated that, in residents, acute SD impaired medical management of life-threatening situations occurring during high fidelity simulated anesthesia cases (Arzalier-Daret et al., 2018). Cognitive deficits emerge and accumulate to significant levels when sleep duration falls below 7 h per night, while fatigue also affects work performance and professional alertness (Saadat et al., 2017). Fatigue in anesthesiologists would have more serious implications that extend beyond individual health and affect patient management, safety, and the quality of healthcare. Saadat et al. (2016) previously reported a significant worsening of mood, the cognitive status, and total mood disturbance after night calls by anesthesiologists compared to normal working hours. While SD has many effects on physicians’ health, this study evaluated the effects of SD on pain thresholds and the redox state of the organism in young male physicians. Studies showed that predictors of pain sensitivity differ by gender (Al’absi et al., 2022), that women experience greater anxiety than men when sleep deprived (Goldstein-Piekarski et al., 2018), and that women are more susceptible to pain in the clinic (Fillingim et al., 2009). Effects of total SD on descending pain inhibition are sex-specific (Eichhorn et al., 2018). Considering the possible influence of gender on the experimental results and the fact that women’s physical conditions are susceptible to menstrual cycles, we selected healthy male residents for the trial. Age was significantly associated with daily sleep duration and subjective sleepiness (Bonnefond et al., 2006), and a previous study demonstrated that somatosensory thresholds for non-injurious stimuli increase with age whereas pressure pain thresholds decrease (Lautenbacher et al., 2005). To reduce the effect of age on the trial results, we included physicians aged 18∼44 years.

In two country samples of community-dwelling older adults in Singapore and Japan, sleep deficiency increased the risk of any new pain onset and pain increased the risk of newly presenting sleep deficiency (Chen et al., 2019). A related clinical study showed that, in healthy adults with mild sleepiness, extended bedtime led to increased sleep duration and decreased drowsiness, resulting in reduced pain sensitivity (Roehrs et al., 2012). To prevent this variation from influencing the results of our experiment, we chose to measure pain thresholds and NRS pain scores at 8:00 a.m. each day. As previously described, we found that one night of SD can lead to significantly higher nociceptive hyperalgesia and NRS pain scores compared to baseline. This was similarly demonstrated in rat experiments, where nociceptive tests on rats SD for 6 days showed that both PWT and PWL were significantly lower in the SDR group compared to the sham group, and this reduction recovered after 4 days of RS. Our study demonstrated that SD leads to increased pain sensitivity in both healthy subjects and animals. Prostaglandin 2 (PGE2), a key mediator of inflammation and pain, has been shown to be a potential mediator in SD-induced changes in pain sensation, and the increase in spontaneous pain caused by total SD is significantly associated with an increase in PGE2 metabolites (Haack et al., 2009). Activation of inflammation-related pathways leads to the production of ROS and RNS, and the generated ROS can lead to the activation of pro-inflammatory cytokines such as IL-1β and TNF-α to induce inflammatory responses, thereby participating in the underlying pathogenic mechanisms of various diseases (Atrooz and Salim, 2020). In this study, it may be related to hyperalgesia in subjects and animals.

The current study showed that SD leads to alterations in the redox state of the body. A recent meta-analysis showed that sleep disturbance is associated with an increase in markers of systemic inflammation (Irwin et al., 2016). Systemic inflammation resulted in a lower pain threshold (de Goeij et al., 2013). It has been shown that SD leads to increased oxidative DNA damage and dying cells in rats and that 2 days of RS restores the balance between DNA damage and repair, leading to normal or below normal oxidative damage (Everson et al., 2014). Sleep is known to replenish redox metabolites in the body, and altered sleep patterns may limit this ability. As expected, in the present study, we found that plasma antioxidant GSH levels and SOD activity decreased with increased oxidative damage, while CAT activity and the MDA levels increased with increased oxidative damage. Thus, we found that antioxidants and redox metabolites in the organism are altered under the influence of SD. The alteration caused by SD is important, especially because previous studies showed that this change is an important factor in neurological diseases (Durmer and Dinges, 2005; Goel et al., 2009; Lowe et al., 2017). It follows that we should not ignore the damage that SD can cause to our body.

The hyperalgesic effect of SD seems reversible by napping (Faraut et al., 2015) as well as by the effective treatment of sleep disorders (Khalid et al., 2011). Roehrs et al. (2012) showed that extended sleep reduces pain sensitivity. Low vigilance following SD might lead to higher pain sensitivity and prevent adequate pain coping, whereas the restoration of vigilance following RS might be accompanied by a normalization of pain perception (Stroemel-Scheder et al., 2020). Several research have shown that RS (i.e., improved quality or quantity of sleep) has the potential to restore normal nociceptive sensitivity after SD-induced nociceptive hypersensitivity (Roehrs et al., 2012; Karmann et al., 2014; Vitiello et al., 2014; Smith et al., 2015). Consistent with the above, our study demonstrated that one night of restorative sleep not only reduces nociceptive hyperalgesia induced by SD but also substantially restores subjects’ subjective pain NRS scores to their pre-SD levels.

Studies related to the effect of RS on altered nociception after SD have been reported, but little has been mentioned about whether RS can improve the altered oxidative stress state of an organization induced by SD. We found that, after one night of RS, MDA levels and CAT vitality could be partially restored, with MDA levels still elevated compared to baseline but significantly lower than after one night of SD and CAT vitality largely restored to baseline levels. Unfortunately, GSH levels and SOD activity were not improved after RS. This shows that even just one night of SD can have a profound effect on redox metabolites. Chronic sleep restriction or acute total SD results in persistent and distinct biological, physiological, and/or neurological changes that are not significantly reversed with chronic, long-duration RS (Yamazaki et al., 2021). A recent study showed that induced sleep deficiency causes the deterioration of body functions and that 1 week of recovery is not enough for full recovery (Ochab et al., 2021). Thus, one night of RS can only partially ameliorate the oxidative damage caused by SD.

Numerous studies were conducted to elucidate the mechanisms of neuropathic pain, and oxidative stress is considered to be one of the important causative factors in damaging peripheral sensory neurons (Areti et al., 2014). Shim et al. (2019) found that the chemotherapeutic drug-induced mechanical hypersensitivity was fractionally mediated by sustained oxidative stress. Indeed, antioxidants can reduce chemotherapy-induced neuropathic pain that has developed in animal models, suggesting that reducing oxidative stress is a promising pain therapy (Kim et al., 2010, 2016; Fidanboylu et al., 2011). We found that SD can cause the body to suffer from oxidative stress and lead to mechanical hypersensitivity. Many studies showed that oxidative stress can cause pain, but whether this mechanical hypersensitivity response is directly mediated by oxidative stress needs further experimental verification. One night of RS can significantly improve mechanical hypersensitivity and only slightly improve oxidative stress damage to the body. It is known that sleep can replenish antioxidants in the body and thus improve the oxidative stress status of the organism. In the present study, the oxidative stress status of the body did not improve well after RS, which may be due to insufficient sleep time or the fact that one night of RS could not compensate for the lack of sleep caused by one night of SD.

We performed proteomic assays in mPFC of sham and SDR rats; overall, we quantified 3,790 proteins using the LFQ proteomics approach, with 2,956 quantifiable proteins. In total, 37 of these proteins were significantly differentially expressed, of which 24 were upregulated and 13 were downregulated during SD. These differentially expressed proteins were mainly involved in biological processes such as starch and sucrose metabolism, amino and nucleotide sugar metabolism, and protein processing in the endoplasmic reticulum. For example, sleep fragmentation induces endoplasmic reticulum stress in the hypothalamus of mice (Hakim et al., 2015), and circadian rhythm dysregulation induced by chronic night work promotes endoplasmic reticulum stress activation, which directly affects the human metabolism (Ferraz-Bannitz et al., 2021).

We acknowledge several study limitations. We did not use electroencephalography (EEG) to monitor our subjects’ sleep conditions during a whole night of SD, so we cannot be sure that they did not experience transient sleep during this time period. However, our subjects were continuously monitored and clinically engaged throughout the night of SD, so we are confident that the subjects did not experience transient sleep during SD. High-intensity and stressful night shift work might be different from simple SD, but we did not study it. Since anesthesiologists work during night shifts, we were unable to perform simple SD on the participating anesthesiologists, which was a limitation of this study. Our study only demonstrated that SD can cause nociceptive hypersensitivity and altered oxidative stress status in the body. Future studies are encouraged to investigate if this nociceptive hypersensitivity is mediated by oxidative stress in patients with sleep-deprived chronic pain.

In conclusion, this study showed that one night of SD can cause nociceptive hypersensitivity and increase oxidative stress damage in the body. One night of RS restored nociceptive hypersensitivity and improved the SD-induced increase in NRS pain scores, but it only slightly improved the markers of oxidative stress after SD. Whether SD-induced nociceptive hypersensitivity is mediated by oxidative stress remains uncertain and further studies are warranted.

## Data availability statement

e original contributions presented in this study are publicly available. This data can be found here: http://www. medresman.org.cn/pub/cn/proj/projectshshow.aspx?proj=4386.

## Ethics statement

The studies involving human participants were reviewed and approved by The First Affiliated Hospital of Zhengzhou University Research Ethics Committee; The First Affiliated Hospital of Zhengzhou University. The patients/participants provided their written informed consent to participate in this study. The animal study was reviewed and approved by The First Affiliated Hospital of Zhengzhou University Research Ethics Committee; The First Affiliated Hospital of Zhengzhou University.

## Author contributions

SC and JYu helped with conceptualization, methodology, analysis, preparation of the manuscript, and review and editing of the manuscript. YX helped with the interpretation of data, methodology, resources, and analysis and was responsible for project administration. YL and XF helped with research design, supervision, interpretation of data, preparation of the manuscript, and approval of the final manuscript. FX helped with data collection and research supervision. YM helped with the conceptualization, methodology, and review and editing of the manuscript. NX and ZW helped with research supervision and review editing. JW helped with the interpretation of data, preparation of the manuscript, and review editing. JYa helped with research design, supervision, and review editing. All authors contributed to the article and approved the submitted version.
